# Rehabilitation of older people with Parkinson’s disease: an innovative protocol for RCT study to evaluate the potential of robotic-based technologies

**DOI:** 10.1186/s12883-020-01759-4

**Published:** 2020-05-13

**Authors:** Roberta Bevilacqua, Elvira Maranesi, Mirko Di Rosa, Riccardo Luzi, Elisa Casoni, Nadia Rinaldi, Renato Baldoni, Fabrizia Lattanzio, Valentina Di Donna, Giuseppe Pelliccioni, Giovanni Renato Riccardi

**Affiliations:** 1 Scientific Direction, IRCCS INRCA, Ancona, Italy; 2 Clinical Unit of Physical Rehabilitation, IRCCS INRCA, Ancona, Italy; 3 Unit of Geriatric Pharmacoepidemiology, IRCCS INRCA, Ancona, Italy; 4 Medical Direction, IRCCS INRCA, Ancona, Italy; 5 Clinical Unit of Physical Rehabilitation, IRCCS INRCA, Fermo, Italy; 6 Neurology Unit, IRCCS INRCA, Ancona, Italy

**Keywords:** Parkinson patients, Gait training, Balance training, Robotic rehabilitation

## Abstract

**Background:**

Parkinson’s disease is one of the most frequent causes of disability among the older adults. It is a chronic-progressive neuro-degenerative disease, characterized by several motor disorders. Balance disorders are a symptom that involves the body axis and do not respond to dopaminergic therapy used in Parkinson’s disease. Therefore, physiotherapy becomes an important intervention for the management of motor disorders. Originally, these rehabilitative approaches were based on empirical experiences, but several scientific evidences suggests that neuronal plasticity is exercise-dependent. In this context, robotic rehabilitation plays an important role because it allows to perform task-oriented exercises and to increase the number of repetitions and their intensity. This protocol study aims to evaluate the effectiveness of robotic-based intervention of the older adults with Parkinson’s disease, designed to improve the gait and to reduce the risk of falling.

**Methods:**

This study is a single-blinded randomized controlled trial. The primary outcomes are: risk of falling, gait performance and fear of falling measured through Performance-Oriented Mobility Assessment (POMA), instrumental gait analysis and Short Falls Efficacy Scale – International (FES-I), respectively. One hundred ninety-five patients with PD will be recruited and randomly divided into three groups, to receive a traditional rehabilitation program or a robotic rehabilitation using Tymo system or Walker View in addition to the traditional therapy. Assessments will be performed at baseline, at the end of treatment and 6 months, 1 year and 2 years from the end of the treatment. A 10-treatment session will be conducted, divided into 2 training sessions per week, for 5 weeks. The control group will perform traditional therapy sessions lasting 50 min. The technological intervention group will carry out 30 min of traditional therapy and 20 min of treatment with a robotic system.

**Discussion:**

The final goals of the present study are to propose a new approach in the PD rehabilitation, focused on the use of robotic device, and to check the results not only at the end of the treatment but also in the long term.

**Trial registration:**

NCT04087031, registration date September 12, 2019.

## Background

Parkinson’s disease (PD) is one of the most frequent causes of disability among the older adults. It is a chronic-progressive neuro-degenerative disease, characterized by motor disorders, such as bradykinesia (poverty and slowness in movement), resting tremor, stiffness, flexed posture and “small steps” gait, not least the balance deficit with consequent high risk of falling [1, 2].

Balance is a fundamental requirement that requires a complex network of sensory information integrations, in order to generate an appropriate motor response aimed at controlling body movement. The balance disorder occurs later during the PD and is a symptom that involves the body axis. It can be highlighted when the person walks or changes direction during the journey. Balance disorders do not respond to dopaminergic therapy used in PD. Therefore, physiotherapy becomes an important intervention for the management of motor disorders. The effects of bradykinesia, rigidity, altered proprioception, postural instability, freezing of the gait, in PD patients, are studied and known in rehabilitation [3–5]. The reduction of balance is considered a risk factor for falls [6–8]. Postural instability and consequent falls are among the main factors that determine the quality of life, morbidity and mortality of a person suffering from PD [3, 5, 7]. The guidelines for physical therapy in the PD recommend that patients undergo a course in preventing falls from the onset of the disease [5]. These pathological conditions are routinely treated through rehabilitative approaches aimed at improving the static and dynamic balance, the recovery of walking and the falls prevention [2, 3, 5–7, 9]. Originally, these rehabilitative approaches were based on empirical experiences, but several scientific evidences suggests that neuronal plasticity is “exercise-dependent” and constitutes the main mechanism that supports the effectiveness of physiotherapy treatment [9–13]. Recent reviews show that rehabilitation induces important clinical benefits particularly in walking speed and balance [9, 10, 12, 13].

In fact, exercise increases synaptic strength and influences neurotransmission, thus potentiating functional circuitry in PD patients, through mechanisms that include increased synaptic strength resulting from raised dopamine and glutamate neurotransmission within the basal ganglia accompanied by increased dendritic spine [14, 15]. Exercise training was also found to produce functional changes (assessed by means of functional Magnetic Resonance Imaging) in known motor learning related brain structures, with aerobic exercise training-dependent neuroplasticity in the central nervous system due to neurochemical changes in the striatum [16]. Synaptic plasticity, neurogenesis and increased cortical vascularization, are three proposed indirect mechanisms underlying physical training-related improvements in PD patients [17, 18]. Moreover, an increased corticomotor excitability [19] and an optimization of the medication intake by easing its absorption [17] may represent direct effects of the improvements seen after exercise training.

In this context, robotic rehabilitation performs an important role [10–12, 20–22]. Different studies [9–11] show that robotic balance training produces performance improvements that are also correlated with evident neurobiological changes in the cerebral cortex [9, 10].

In a recent systematic review and meta-analysis robot-assisted gait training (RAGT) showed better outcomes than conventional interventions on some motor aspects in PD [23], and greater advantages than treadmill training for individuals with freezing of gait-related disability were observed in another study [24]. Upper limb motor performances in patients with PD were also improved after motor training aided by high technological devices [25].

Moreover, there is increasing evidence that suggest the use of the latest computerized technologies could revolutionize conventional treatments and provide better rehabilitation in patients with neurological diseases such as PD, by providing safe and objective real-time assessments of behavior [26].

Thus, thanks to the opportunity offered by technology of providing and controlling the provision of repetitive training/tasks/exercises, that represents the key factor for successful rehabilitation strategies, we hypothesize that an improvement of the primary end point (balance and gait) may be obtained through the use of the systems.

### Study aims and objective

This protocol study aims to evaluate an innovative rehabilitation treatment based on robotics, for the older patients with Parkinson’s disease, designed to improve the gait and to reduce the risk of falling. The treatment involves the use of two robotic devices: Tymo system (Tyromotion, Austria), a wirelesss static and dynamic platform, for evaluating and rehabilitating posture, and Walker View (TecnoBody, Italy), a treadmill equipped with a sensorised belt with eight load cells and a 3D camera.

The primary aim is the evaluation of the effect of the rehabilitation treatment on the balance and gait of the older PD patient, as a result of the use of the Tymo system or Walker View, at the end of the treatment and at a 6 months, 1 and 2 years’ follow-up using POMA scale.

The secondary aims are:

1. the evaluation of the effect of the rehabilitation treatment on the gait speed of the older PD patients, as a result of the use of the Tymo system or Walker View, at the end of the treatment and at a 6 months, 1 and 2 years’ follow-up using instrumental gait analysis;
2. the evaluation of the effect of the rehabilitation treatment on the fear of falling of the older PD patients, as a result of the use of the Tymo system or Walker View, at the end of the treatment and at a 6 months, 1 and 2 years’ follow-up using FES-I short form scale.

In addition, the study design will include use a standardized questionnaires and instrumental gait analysis, in order to collect data on the improvements with a mix-method approach.

## Methods/design

### Trial design

This study is a single blinded (outcome assessors) randomized controlled trial. One hundred ninety-five patients with PD will be recruited and randomly divided into three groups, to receive a traditional rehabilitation program or, in addition to the traditional therapy, a robotic rehabilitation using Tymo system or Walker View. Assessments will be performed at baseline, at the end of treatment and 6 months, 1 year and 2 years from the end of the treatment. For the duration of the study, patients cannot receive treatments through robotic devices but will continue to be followed as outpatients by our rehabilitation department.

### Study setting

The study is conducted at the Clinical Unit of Physical Rehabilitation, IRCCS INRCA, in the Ancona and Fermo branches, Italy. Assessments and treatments are conducted in the robotics laboratories.

### Trial status

At the time of the submission of this study protocol, data collection is ongoing.

### Participants

The inclusion criteria are:

- Aged 65 and over;
- Capacity to consent;
- Hoen and Yahr scale: 1–3 stage;
- Functional Ambulation Category (FAC) ≥ 2;
- Ranking scale score ≤ 3;
- Stability of drug treatment for at least 1 month;
- Geriatric Depression Scale 5-items: negative;

The exclusion criteria are:

- Failure to meet the inclusion criteria;
- Concomitant participation in other studies;
- Lack of written informed consent;
- Clinical dementia rating (CDR) score ≥ 3;
- History of syncopal episodes, epilepsy and vertigo not controlled pharmacologically;
- Serious dysfunction of the autonomic system;
- Severe behavioral syndromes not compensated by drugs;
- Concurrent neurological diseases;
- Severe systemic diseases with life expectancy < 1 year;
- Patients unable to follow up.

### Sample size

The Performance-Oriented Mobility Assessment (POMA) [27], a test widely used to assess walking ability and associated with equilibrium, is used to calculate the sample size [28]. Assuming a small effect size of 10% [29], it is estimated that the overall sample size needed to capture this effect size is of 153 subjects, assuming a statistical power of 80%, a significance level of 0.05, three groups and 5 repeated assessments (a baseline and 4 follow-ups) in an ANOVA model within-between interactions. Even assuming a 25% drop out rate, the total number required would be 195 subjects (65 for each arm).

It is hypothesized that this sample dimension is more than sufficient to grasp a variation also for secondary outcomes for which a treatment effect size is assumed of a similar or higher entity than that identified for the primary outcome [30, 31].

### Recruitment

Patients are selected by the outpatient department at the Clinical Unit of Physical Rehabilitation, IRCCS INRCA, in the Ancona and Fermo branches. These patients are contacted to schedule a visit with the physician. Once the compliance with the inclusion and exclusion criteria of the study has been verified and the informed consent (see Supplementary file) has been obtained in triplicate, the doctor proceeds with the baseline evaluation and with acquisition of gait assessment parameters through Gait Analysis at the Movement Analysis Laboratory of the Clinical Unit of Physical Rehabilitation of the Ancona branch.

A randomization technique based on a single sequence of random assignments is used. A list of random numbers generated by the computer is used and subject is assigned a number based on their order of inclusion in the study. According to this technique, the 195 subjects are randomly assigned to one of the 3 study groups.

At the end of the treatment and after 6 months, 1 year and 2 years, the patients will be contacted again to schedule subsequent follow-up visits and upgrades. During the follow-up period, patients will be monitored through telephone interviews once a month, in order to monitor their health state.

Recruitment will run from August 2019 to August 2021.

### Intervention

For the study, post-hospitalization subjects are taken into consideration, after 4 weeks from the hospitalization in the Clinical Unit of Physical Rehabilitation, IRCCS INRCA, in the Ancona and Fermo branches, after having already received the standard treatment. A 10-treatment session is conducted, divided into 2 training sessions per week, for 5 weeks. The control group performs traditional therapy sessions lasting 50 min. The technological intervention group, using Tymo system or Walker View, carries out 30 min of traditional therapy and 20 min of treatment with a robotic system. Cardiac activity monitoring is planned during robotic treatments in order to detect the heart rate during physical activity [32].

Individual participants must complete at least 80% of the sessions. Recovery of 2 sessions will be possible.

All patients included in the study perform traditional rehabilitation treatments, consisting in:

- Breathing, relaxation and postural harmonization exercises;
- Exercises of active mobility and stretching;
- Eye-hand coordination exercises and couple exercises;
- Exercises for walking and verticalization.

The robotic treatment consists of using two different devices: Tymo system or Walker view.

Tymo system is a wireless platform for the balance and the postural control training. Tymo system is connected to a screen and provides virtual reality games, adaptable to the functional capacity of the patient. Through the games proposed, the physiotherapist decides to work in a dimension (antero-posterior or medio-lateral dimension) or in two dimensions (combining the antero-posterior and medio-lateral movements).

Walker view is a treadmill equipped with a sensorized belt with eight load cells and a 3D camera to detect length, speed and symmetry of the pace and load, range of the trunk, hips and knees. Patients are asked to walk at a comfortable speed, while the physiotherapist is able to work on different parameters such as step length, load distribution, and step height. The setting takes place taking into account the clinical conditions of each patient, customizing the intervention. The Walker View offers visual and auditory feedback to the patient, so as to correct gait in real time.

Adverse events, although unlikely, could be related to the use of technology such as falls and/or pain at knees or ankles.

### Outcomes

All outcome measures follow a standardized operating procedure. Table 1 shows the primary outcome and the secondary outcomes with the expected result at the end of the treatment. The expected improvement was derived from the analysis of similar studies [29], collected for the evaluation of the sample size for each outcomes.

**Table 1:** Outcomes and clinical assessments

Outcome(s)	Clinical assessment	Expected improvement at the end of treatment
Primary: Improvement of the overall mobility (balance + walking ability)	POMA	10%
Secondary: Improvement of gait speed	Instrumental Gait analysis	12%
Secondary: Decrease of fear of falling	FES-I Short form	15%

*POMA* Performance-Oriented Mobility Assessment, *FES-I* Short Falls Efficacy Scale – International

Further evaluations are carried out as follows:

- Length and asymmetry of the step, through instrumental Gait Analysis;
- Walking and functional status through the Functional Ambulation Category and Barthel Index scale;
- Acceptance of the technology, through Psychosocial impact of assisted device scale (PIADS) questionnaire
- Quality of life, through SF-12 questionnaire.

#### Mini-mental state examination (MMSE)

The Mini-Mental State Examination was designed as a clinical method for grading cognitive impairment. The score ranges from 0 to 30: scores ≥24 indicate normality, between 18 and 23 indicate mild cognitive impairment, between 11 and 17 average cognitive deficits, scores ≤10 severe cognitive impairment. The reported score is corrected for age and education [33].

#### Rankin scale (RA)

It is a simple scale for the evaluation of the outcomes following the stroke. Reliability is well defined. The individual categories are essentially based on patient mobility. There are 6 grades of classification from 0 to 5, where 0 means independence [34].

#### Hoehn and Yahr scale (SHY)

This scale is used in the medical field to describe the symptoms of Parkinson’s disease progression. It was originally published in 1967 in the Neurology Journal by Melvin Yahr and Margaret Hoehn, and included stages 1 to 5. Since then, a modified scale has been proposed, with the addition of stages 1.5 and 2.5 describing the intermediate course of the disease [35].

#### Barthel index (BI)

BI is an ordinal scale used to measure a subject’s performance in everyday life activities. The index analyzes ten variables that describe the activities of daily life and mobility. Each item is assigned a score between 0 and 10 depending on the degree of patient’s functionality: full, reduced or no functionality. A high overall score is associated with a greater probability of being able to live at home independently after discharge from the hospital [36].

#### Functional ambulation categories (FAC)

The scale is used to classify the severity level of gait disturbances in neurological disorders. It provides a hierarchical classification from level 0 (impossible walking) to level 5 (no limitation) [37].

#### SF-12 health survey (SF-12)

The SF-12 questionnaire was originally developed in the United States to provide a short alternative form to the SF-36 questionnaire. The SF-12 is composed of 12 items that produce two measurements related to two different aspects of health: physical health and mental health. The subject is asked to answer on how he feels and how he is able to carry out the usual activities, evaluating the current day and the 4 previous weeks [38].

#### Tinetti’s scale or performance-oriented mobility assessment (POMA)

The Tinetti scale is a tool used to evaluate balance and gait performance. The test is used clinically to determine the mobility status of a subject or to assess changes in balance and gait time. The total POMA (POMA-T) consists of two sub-scales: the balance evaluation scale (“balance scale” or POMA-B) and the gait evaluation scale (“gait scale” or POMA-G). the maximum score is 28 points: in detail, the maximum score of the POMA-B is 16, while for the POMA-G the maximum score is 12 [27].

#### Short falls efficacy scale - international (FES-I- short)

The scale measures the “fear of falling”. The scale can be self-administered or administered during the interview. The cut offs for the fear of falling are divided as follows: a score between 7 and 8 indicates a low concern, between 9 and 12 a moderate concern and between 14 and 28, high concern [39].

#### Psychosocial impact of assistive devices scale (PIADS)

It is a self-completed questionnaire by the user and it assesses the impact that the device has on the person. Through 26 questions it tries to detect how the device has brought about a perception of change with respect to one’s availability for new experiences (6 questions), skills (ability to cope with daily activities and challenges - 12 questions) and self-esteem (security and self-confidence - 8 questions). Every question is answered on a visual scale marked by − 3 (the device has strongly limited my independence) to + 3 (the device has greatly improved my independence) [40].

#### Assistive device predisposition assessment (ATDPA)

The purpose of the tool is to assess user expectations about technological devices [41].

#### Geriatric depression scale 5-items version (GDS)

This questionnaire assesses the current condition of the patient’s mood. For the screening required by our study, only the first five items of the scale can be used. The answers highlighted indicate the statements expected by a non-depressed subject [42].

#### Clinical dementia rating scale (CDR)

This questionnaire assesses the patient’s dementia status. The CDR is a 5-point scale used to characterize six domains: memory, orientation, judgment and problem solving, business, home and hobby and personal care [43].

#### Gait analysis and instrumental postural analysis

Gait analysis is the systematic study of human locomotion, augmented by instrumentation for measuring body movements, body mechanics, and the activity of the muscles [44]. Gait analysis is performed on the selected patients at the Gait Analysis Laboratory in the Department of Physical Rehabilitation at branch of IRCSS INRCA Ancona. Instrumented gait analysis is performed using BTS GAITLAB (BTS Bioengineering, Italy) system with six infrared cameras (100 Hz) and 2 force plates (50 Hz). The system is used to acquire both kinematic and kinetic data. Three-dimensional kinematic data are recorded with the help of 22 reflective infrared markers using Helen Hayes protocol [45]. The floor-mounted force plates are used to acquire the kinetic data. The subjects walk at a self-selected speed. The instrumental postural analysis studies the complex control system that must keep the center of gravity constantly in a balanced position. This analysis is carried out through an advanced system consisting composed by a camera for video recording and a platform with 2 triaxial sensory plates (Podium, BTS Bioengineering, Italy). With this system, it is possible to simultaneously visualize the three components of force: vertical, antero-posterior and lateral. It allows to evaluate, in augmented reality, the symmetry of the load and the trend of the pressure center.

A summary of all data collected and when these are collected is provided ion Table 2.

**Table 2:** Schedule of assessment and outcome measures

Outcome	Clinical assessment	R	T1	FW1	FW2	FW3
Cognitive State	Mini Mental State Examination	✓				
Gait parameters	Functional Ambulation Category	✓	✓	✓	✓	✓
Disability State	Rankin Scale	✓				
Cognitive State	CDR	✓				
Functional State	Barthel Index	✓	✓	✓	✓	✓
Parkinson State	Hoehn and Yahr Scale	✓				
Quality of life	SF-12 Health Survey	✓	✓	✓	✓	✓
Sociodemographic characteristics	Check-list	✓				
Motor ability	Motricity index	✓	✓	✓	✓	✓
Depression State	Geriatric Depression scale 5-items version	✓				
Attitude to technology	Assistive Device Predisposition Assessment – Scala E	✓				
Fall risk	Scala di Tinetti	✓	✓	✓	✓	✓
Gait parameters	Gait Analysis + instrumental postural analysis	✓	✓	✓	✓	✓
Fear of falling	Short Falls Efficacy Scale - International	✓	✓	✓	✓	✓
Acceptance of technology	Psychosocial impact of assisted device scale - PIADS		✓			

*R* Recruitment, *T1* end of treatment, *FW1* first follow up at 6 months since the end of treatment, *FW2* second follow up at 1 year since the end of treatment, *FW3* third follow up at 2 years since the end of treatment

### Data management

Personal data collected during the trial will be handled and stored in accordance with the General Data Protection Regulation (GDPR) 2018. Use of the study data will be controlled by the principal investigator. All data and documentation related to the trial will be stored in accordance with applicable regulatory requirements and access to data will be restricted to authorized trial personnel.

### Data analysis

Continuous variables will be reported as either mean and standard deviation (SD) or median and interquartile range (IQR) on the basis of their distribution (assessed using Shapiro-Wilk test). Comparison of variables between groups will be performed by unpaired Student t test or Mann-Whitney U test according to their distribution. Categorical variables will be expressed as absolute number and percentage and statistical significance will be assessed by Chi-square test or Fisher Exact.

In a second step, the analysis of the follow-up data will be carried out in order to evaluate the effectiveness of the intervention. This analysis phase will involve the use of multivariate statistical techniques, in particular repeated measures analysis of variance (ANOVA), in order to compare the changes over time in the outcome measures between the intervention group and the control group. The statistical significant will be set at *p* < 0.05.

## Discussion

The aim of the present study protocol is to evaluate an innovative rehabilitation treatment of the older adults with Parkinson’s disease, designed to improve the gait and to reduce the risk of falling. This innovative rehabilitation program involves two different robotic devices that work using visual or auditory feedback in order to correct wrong postural or gait patterns. Moreover, further evaluations, such as length and asymmetry of the step, walking and functional status and acceptance of the technology will be carried to underline the efficacy of this innovative treatment.

The study population will be given different questionnaires (Mini-Mental State Examination, Rankin scale, Barthel Index, Functional Ambulation Categories, Hoehn and Yahr Scale, SF-12 Health Survey, Tinetti’s Scale, Motricity index, Short Falls Efficacy Scale – International, Psychosocial Impact of Assistive Devices Scale, Assistive Device Predisposition Assessment, Geriatric Depression scale 5-items version, Clinical Dementia Rating Scale, General Data Protection Regulation) that take into consideration different aspects of the patient’s health status.

We focus on PD patients with mild subjective cognitive complaints that are expected to have the opportunity to employ significant neural plasticity in response to motor rehabilitation program with robotic devices. In order to analyze between-group differences in control group, treated only with traditional rehabilitation, and experimental groups, treated with robotic devices (Tymo system or Walker View), we will randomize the included patients to have results as reliable and error-free as possible.

Another important aspect of this protocol is the check of the results obtained not only at the end of the treatment but also in the long term, foreseeing 3 follow-up, at 6 months, 1 year and 2 years from the end of the rehabilitation program.

The dissemination program will involve peer-reviewed journal and national and international conferences. Moreover, the results will be disseminated to all participants.

## Supplementary information

**Additional file 1.**

## Data Availability

The datasets generated, used and analyzed during the trial and its preceding pilot trial are or will be available from the corresponding author upon reasonable request.

## Abbreviations

ATDPA: Assistive Device Predisposition Assessment
BI: Barthel Index
CDR: Clinical Dementia Rating Scale
FAC: Functional Ambulation Categories
FES-I-Short: Short Falls Efficacy Scale - International
GDPR: General Data Protection Regulation
GDS: Geriatric Depression scale 5-items version
MI: Motricity index
MMSE: Mini-Mental State Examination
PD: Parkinson’s disease
PIADS: Psychosocial Impact of Assistive Devices Scale
POMA: Tinetti’s Scale or Performance-Oriented Mobility Assessment
RA: Rankin scale
SF-12: SF-12 Health Survey
SHY: Hoehn and Yahr Scale
