# Supplementary material for: Rehabilitation of older people with Parkinson’s disease: an innovative protocol for RCT study to evaluate the potential of robotic-based technologies
Source: BMC Neurol. 2020 May 13;20:186. doi: 10.1186/s12883-020-01759-4 (PMC7222584; doi:10.1186/s12883-020-01759-4)
Supplement: Supplementary file 1 — Additional file 1. [file 12883_2020_1759_MOESM1_ESM.docx]

# MODULO DI CONSENSO INFORMATO SCRITTO

**TITOLO DELLO STUDIO: ROBOTEC Lab - La ROBOtica assistiva nell’anziano: modelli innovativi nella riabilitazione dell’anziano con malattia di Parkinson attraverso l’innovazione TECnologica**

**CODICE DELLO STUDIO:**

**COGNOME E NOME DEL RESPONSABILE DEL PROGETTO: Giovanni Renato Riccardi**

**COGNOME E NOME DELL’INTERVISTATORE: _________________________**

Io sottoscritto/a (nome e cognome) __________________________________________

Età ________ sesso M ⬜ F ⬜ data di nascita ___/___/_____

Indirizzo: Via/Piazza ______________________________n. _______ CAP _________

Città _________________________ tel. _______________________

**Dichiaro di:**

• partecipare volontariamente alla sperimentazione in oggetto, avente come scopo principale la valutazione di un innovativo trattamento riabilitativo dell’anziano con malattia di Parkinson, volto al miglioramento dell’andatura e dell’equilibrio. Il trattamento prevede l’utilizzo del *Tymo System*, un dispositivo wireless utilizzato per il training dell’equilibrio e del controllo posturale e del *Walker view*, un treadmill dotato di nastro sensorizzato a celle di carico che permette la valutazione degli appoggi durante la deambulazione che, grazie all’interfaccia software, corregge in tempo reale i parametri dinamici. In seguito al trattamento standard, sarà condotto un allentamento di 10 sessioni, suddivise in 2 allenamenti a settimana, per la durata totale di cinque settimane.

• aver ricevuto dall’intervistatore/ricercatore sopra menzionato tutte le informazioni chiare ed esaurienti sulle finalità e le procedure a cui mi è stato chiesto di prendere parte;

• aver letto e compreso il foglio di informazioni che mi è stato consegnato con sufficiente anticipo e che conferma quanto mi è stato verbalmente detto;

• aver avuto l’opportunità di porre domande chiarificatrici e di aver avuto risposte soddisfacenti, come pure di aver avuto la possibilità di informarmi sui particolari dello studio con persona di mia fiducia;

• essere stato/a informato/a sui possibili benefici che potrei trarre e sui rischi o disagi ragionevolmente prevedibili, e di aver avuto il tempo sufficiente per decidere;

• essere consapevole:

– che i miei dati potranno essere esaminati o utilizzati per fini di ricerca, ma resteranno strettamente riservati nel rispetto della normativa vigente e successive modifiche e integrazioni;

– che i miei dati saranno utilizzati in forma aggregata, per la redazione di un rapporto finale destinati alle Autorità Sanitarie o ad una pubblicazione, qualunque sia l’esito dello studio, sempre nel rispetto della confidenzialità e anonimato della mia identità (Regolamento (UE) 2016/679 del Parlamento europeo e del Consiglio, del 27 aprile 2016*,* art. 13 del DLgs n. 196/03 in vigore dal 1° gennaio 2004);

– di dover firmare due moduli identici del presente consenso informato: un originale verrà trattenuto dall’intervistatore/ricercatore (e conservato per almeno 15 anni) e il secondo mi verrà consegnato;

– che per ogni problema o per eventuali ulteriori informazioni dovrò rivolgermi a:

Nome e Cognome del responsabile Dr. Giovanni Renato Riccardi

*Via della Montagnola, 81, Ancona*

071-8003403

• che nel mio proprio interesse lo sperimentatore potrà decidere il mio ritiro dallo studio.

**Pertanto acconsento liberamente alla partecipazione alla compilazione del questionario**.

La firma su questo modulo non verrà ad incidere sui miei diritti legali.

Letto e approvato (scritto a mano) _______________________________________

**(Facoltativo)** Inoltre acconsento alla ripresa di immagini fotografiche e video concernenti la mia partecipazione allo studio, autorizzando a titolo gratuito, senza limiti di tempo, anche ai sensi del Regolamento (UE) 2016/679 del Parlamento europeo e del Consiglio, del 27 aprile 2016*,* degli artt. 10 e 320 cod.civ. e degli artt. 96 e 97 legge 22.4.1941, n. 633, Legge sul diritto d’autore, alla pubblicazione e/o diffusione in qualsiasi forma delle proprie immagini su carta stampata e/o su qualsiasi altro mezzo di diffusione. Inoltre autorizzo alla conservazione delle foto e dei video stessi negli archivi informatici dei partner del progetto “eWARE”, prendendo atto che la finalità di tali pubblicazioni è meramente di carattere informativo ed eventualmente promozionale del progetto suddetto.

Letto e approvato (scritto a mano) _______________________________________
